# X-Ray Photoelectron Spectroscopy on Microbial Cell Surfaces: A Forgotten Method for the Characterization of Microorganisms Encapsulated With Surface-Engineered Shells

**DOI:** 10.3389/fchem.2021.666159

**Published:** 2021-04-22

**Authors:** Hao Wei, Xiao-Yu Yang, Henny C. van der Mei, Henk J. Busscher

**Affiliations:** ^1^University of Groningen and University Medical Center of Groningen, Department of Biomedical Engineering, Groningen, Netherlands; ^2^State Key Laboratory of Advanced Technology for Materials Synthesis and Processing, Wuhan University of Technology, Wuhan, China; ^3^School of Engineering and Applied Science, Harvard University, Cambridge, MA, United States

**Keywords:** self-assembly, nanobiomaterials, biomineralization, yolk shell, click chemistry, zeta potentials, biosorption, beer brewing

## Abstract

Encapsulation of single microbial cells by surface-engineered shells has great potential for the protection of yeasts and bacteria against harsh environmental conditions, such as elevated temperatures, UV light, extreme pH values, and antimicrobials. Encapsulation with functionalized shells can also alter the surface characteristics of cells in a way that can make them more suitable to perform their function in complex environments, including bio-reactors, bio-fuel production, biosensors, and the human body. Surface-engineered shells bear as an advantage above genetically-engineered microorganisms that the protection and functionalization added are temporary and disappear upon microbial growth, ultimately breaking a shell. Therewith, the danger of creating a “super-bug,” resistant to all known antimicrobial measures does not exist for surface-engineered shells. Encapsulating shells around single microorganisms are predominantly characterized by electron microscopy, energy-dispersive X-ray spectroscopy, Fourier transform infrared spectroscopy, particulate micro-electrophoresis, nitrogen adsorption-desorption isotherms, and X-ray diffraction. It is amazing that X-ray Photoelectron Spectroscopy (XPS) is forgotten as a method to characterize encapsulated yeasts and bacteria. XPS was introduced several decades ago to characterize the elemental composition of microbial cell surfaces. Microbial sample preparation requires freeze-drying which leaves microorganisms intact. Freeze-dried microorganisms form a powder that can be easily pressed in small cups, suitable for insertion in the high vacuum of an XPS machine and obtaining high resolution spectra. Typically, XPS measures carbon, nitrogen, oxygen and phosphorus as the most common elements in microbial cell surfaces. Models exist to transform these compositions into well-known, biochemical cell surface components, including proteins, polysaccharides, chitin, glucan, teichoic acid, peptidoglycan, and hydrocarbon like components. Moreover, elemental surface compositions of many different microbial strains and species in freeze-dried conditions, related with zeta potentials of microbial cells, measured in a hydrated state. Relationships between elemental surface compositions measured using XPS in vacuum with characteristics measured in a hydrated state have been taken as a validation of microbial cell surface XPS. Despite the merits of microbial cell surface XPS, XPS has seldom been applied to characterize the many different types of surface-engineered shells around yeasts and bacteria currently described in the literature. In this review, we aim to advocate the use of XPS as a forgotten method for microbial cell surface characterization, for use on surface-engineered shells encapsulating microorganisms.

## Introduction

Life needs continuous protection against environmental conditions. The development of ordered microscopic structures on the surface of archaea has placed them earlier on the evolutionary timeline than bacteria (Wang et al., 2020). In medieval times, knights were harnessed in metal frames to protect them in battle. Environmental conditions necessitating protection have varied over the ages and currently, human life needs protection e.g., against Ultra Violet (UV) irradiation that can be achieved by protecting cells in and underneath the skin by application of UV absorbing creams on the skin.

Protection of life, including microbial life, in most cases starts at the surface. Microorganisms like yeasts and bacteria occur in many different and highly diverse environments. Yeasts and bacteria are useful in several industrial and natural environments. Yeasts are pivotal in the production of alcohol and brewing industry, but do not survive high concentrations of alcohol. Many bacterial strains most notably *Bacillus subtilis*, are used in bioreactors (Jiang et al., 2015a), bio-fuel production (Abalde-Cela et al., 2015), and biosensors (Liu et al., 2009), but here too their application is limited by strong light conditions, extreme pH values and (self-produced) toxins in bioreactors and biosensors. Bacteria play various roles in human health and disease. A healthy human host is said to possess 30 trillion tissue cells and 39 trillion bacteria without whom the oral cavity would be less protected against invading viruses and other microorganisms, the digestive tract would not function properly and human life would be impossible. However, apart from the “healthy” microbial strains and species supporting human life, human life is at the same time threatened by “bad” or pathogenic microorganisms. It is anticipated that by the year 2050 antibiotic-resistant infections will constitute the number one cause of death due to the ongoing increase of antibiotic-resistance amongst human pathogens (Tagliabue and Rappuoli, 2018). Accordingly, “healthy” microbial strains and probiotic bacteria administered through various over-the-counter products, may need protection against the acidic conditions in the gastro-intestinal tract after oral administration or during antibiotic treatment to eradicate infecting pathogens (Anselmo et al., 2016; Li et al., 2018).

Protecting industrially-employed microorganisms and the “good” microorganisms in the human body is an ever-growing field of research and can be done by modifying the genetic code of the organisms or by encapsulating them in surface-engineered shells that interact with the cell surface. Surface-engineered shells are temporary and break upon microbial multiplication, which makes them preferable above genetically-engineered shells that may bear the risk of inducing a “super-bug” resistant to all know antimicrobials.

Encapsulating surface-engineered shells should not only protect, but also allow bidirectional diffusion of molecules, including influx of oxygen, nutrients and growth factors, and outward transport of waste products. Cellular encapsulation by hydrogels has been widely investigated (Uludag et al., 2000) and nowadays extends to nano-engineered shells composed by organic, inorganic, and organic-inorganic hybrid materials.

Application of surface-engineered shells requires precise control of interfacial interactions between the cell surface and the encapsulating shells, the porosity of the shells and the surface properties of the shells that control microbial interaction with their environment. Typically, encapsulating shells around single microorganisms are characterized for their morphology and structure by electron microscopy and X-ray diffraction. Composition is determined by energy-dispersive X-ray spectroscopy and Fourier transform infrared spectroscopy, while particulate micro-electrophoresis is often applied to assess the charge properties of encapsulating shells. Porosity is quantitated using nitrogen adsorption-desorption isotherms. Surprisingly, X-ray Photoelectron Spectroscopy (XPS) is lacking as a technique to characterize the shells applied for microbial encapsulation, despite the fact that XPS has been applied extensively in the past to establish relations between microbial cell surface composition with their physico-chemical properties and function. XPS can be easily applied on microbial cells surfaces after freeze-drying. Freeze-drying leaves the microorganisms intact to form powders that can be pressed in small cups, suitable for insertion in the high vacuum of an XPS machine and obtaining high resolution spectra. Our analysis of the literature dealing with microbial encapsulation and our conclusion that XPS has seldom been applied to characterize encapsulating shells around microorganisms, has stimulated us to use the sub-title “*a forgotten method for the characterization of microorganisms encapsulated with surface-engineered shells*” in the title of this review.

With the aim of bringing the XPS community together with the highly multi-disciplinary research community of microbial cell encapsulation, we firstly provide a brief overview of the most common types of surface-engineered shells. Secondly, we extensively describe the preparation of microbial samples for XPS analyses, together with important results obtained using microbial cell surface XPS. Finally, selected examples are presented on XPS characterization of encapsulating shells around different microorganisms.

## Overview of Different Surface-Engineered Encapsulating Shells and Their Characterization

Many different types of surface-engineered shells have been described in the literature, that can be classified based on the encapsulating material employed (Table 1). Also, different characterization methods have been employed to study the physico-chemical properties of the encapsulated microorganisms. Herein we summarize the most common encapsulation methods and frequently used characterization methods, with the aim of introducing surface-engineered microbial encapsulation methods to the XPS community rather than presenting a full, comprehensive summary of all encapsulation methods.

**Table 1:** Overview of the most common, different encapsulation, and characterization methods applied, organized according to the type of encapsulating material employed.

**Encapsulating material**	**Encapsulation method**	**Schematic**	Characterization methods applied*	**References**
Organic	Layer-by-layer self-assembly	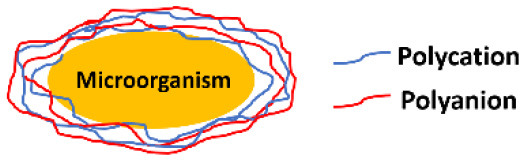	SEM, TEM, CLSM, FTIR, NMR, Zeta potentials	Diaspro et al., 2002; Kozlovskaya et al., 2011; Drachuk et al., 2012; Eby et al., 2012; Anselmo et al., 2016
	Mussel-inspired dopamine self-polymerization	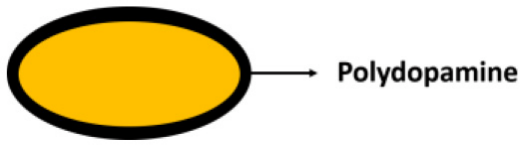		Yang et al., 2011; Su et al., 2019
	Ligand-receptor binding	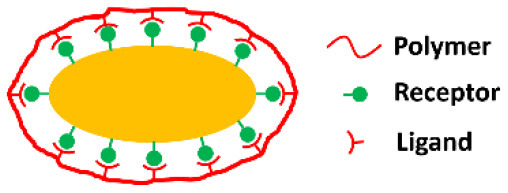		Zheng et al., 2020
Inorganic	Direct deposition	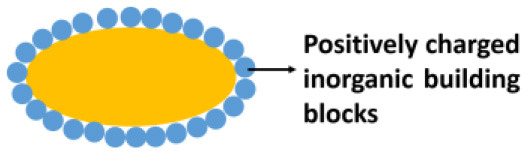	SEM, TEM, CLSM, FTIR, NMR, Zeta potentials, XRD, Nitrogen adsorption-desorption	Berry et al., 2005; Kempaiah et al., 2011
	Click chemistry	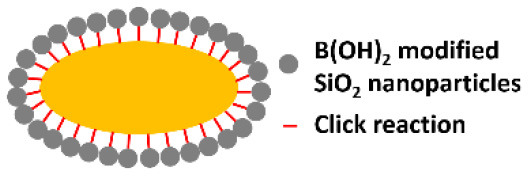		Geng et al., 2019
	Yolk shell	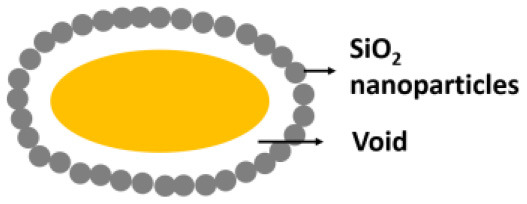		Wang et al., 2020
	Biomineralization	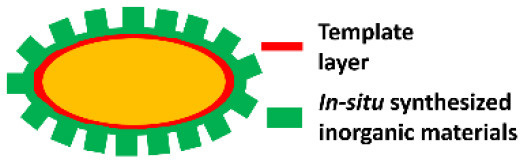		Wang et al., 2008; Yang et al., 2009, 2012; Ko et al., 2013
Organic-inorganic hybrid	Layer-by-layer self-assembly	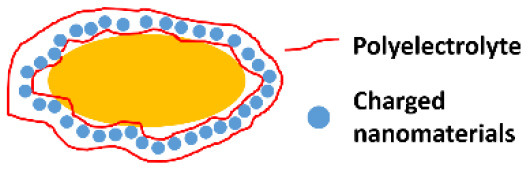	SEM, TEM, FTIR, Zeta potentials, XRD	Fakhrullin et al., 2010; Wang et al., 2010; Zamaleeva et al., 2010
	Bio-interfacing	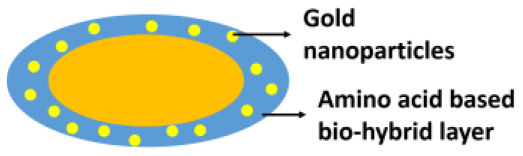		Jiang et al., 2014, 2015a,b
	MOF biomineralization	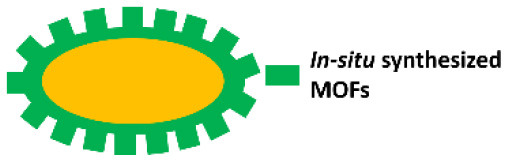		Park et al., 2014; Liang et al., 2016

* *SEM, scanning electron microscopy; TEM, transmission electron microscopy; CLSM, confocal laser scanning microscopy; FTIR, Fourier transform infrared spectroscopy; NMR, nuclear magnetic resonance spectroscopy; XRD, X-ray diffraction*.

### Organic Encapsulating Materials

**Figure 1:**
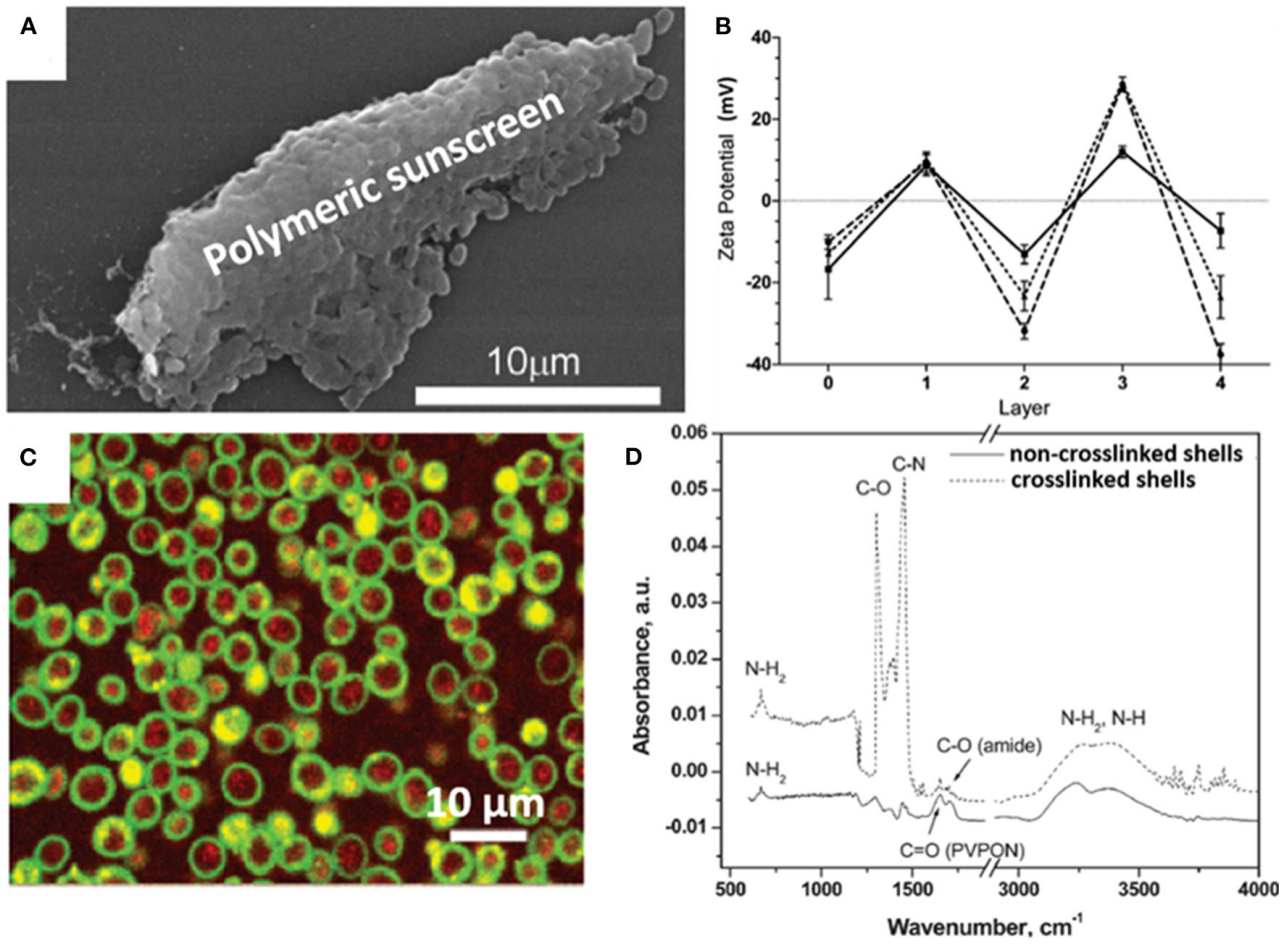
Examples of characteristics of different organic, surface-engineered shells. **(A)** SEM micrograph of *Escherichia coli* encapsulated by layer-by-layer self-assembly of cationic polyallylamine and anionic humic acid for UV-protection (Eby et al., 2012, copyright 2012, American Chemical Society). **(B)** Zeta potentials of *E. coli* upon self-assembly of different layers of cationic polyallylamine (layers 1 and 3) and different anionic polyelectrolytes (layers 2 and 4), including poly(vinyl sulfate) (solid line), poly(styrenesulfonate) (dashed line), and humic acid (dotted line) (Eby et al., 2012, copyright 2012, American Chemical Society). **(C)** Layer-by-layer, polyelectrolyte encapsulated *S. cerevisiae* observed using CLSM. Red-fluorescence indicates metabolically active DAPI stained yeasts, while green-fluorescence represents the polyelectrolyte shell (Diaspro et al., 2002, copyright 2002, American Chemical Society). **(D)** FTIR spectra of (PMAA-co-NH_2_)_5_ layer-by-layer self-assembled shells on *S. cerevisiae* surfaces before and after cross-linking to create a robust shell (Drachuk et al., 2012, copyright 2012, American Chemical Society).

Organic materials can be applied to encapsulate microbial cell surfaces using layer-by-layer self-assembly, self-polymerization or ligand-receptor binding (see Table 1). Layer-by-layer self-assembly is achieved by sequential adsorption of oppositely charged molecules on microbial surfaces, established mainly by electrostatic double-layer attraction (Fakhrullin and Lvov, 2012) and hydrogen bonding (Kozlovskaya et al., 2011). Polyelectrolytes, amino acids and proteins have all been applied for encapsulating bacteria (Eby et al., 2012; Anselmo et al., 2016) and yeasts (Diaspro et al., 2002) using layer-by-layer self-assembly. Self-assembled layers of cationic polyallylamines and different anions (Figure 1A) on *Escherichia coli* surfaces acted as a “sun-screen” for the bacteria against UV-light and demonstrated the typical alternating positive-negative zeta potential pattern upon application (Figure 1B), characteristic in layer-by-layer self-assembly. Strong bonding may affect the viability of the encapsulated microorganisms, depending on the strain and encapsulating material applied and can be assessed using fluorescent staining and confocal laser scanning microscopy (CLSM) (Figure 1C). Importantly, self-assembled layers have also been described to act as a template for further encapsulation. For example, (PDADMAC/PSS)_6_-PDADMAC self-assembled layers on *Synechocystis* were used as a template for further biomimetic silicification (Xiong et al., 2013) and (PDADMAC/PAA)_4_ layers on *Saccharomyces cerevisiae* were applied for subsequent encapsulation by calcium phosphate (Wang et al., 2008). pH-responsive, poly(methacrylicacid) nanoshells brought on by a layer-by-layer method and subsequent cross-linking (Figure 1D), were used on *S. cerevisiae* surfaces to manipulate their growth kinetics in response to environmental pH changes (Drachuk et al., 2012).

Unlike layer-by-layer self-assembled encapsulating shells, mussel-inspired dopamine self-polymerized shells strongly interact with microbial cell surfaces, predominantly through covalent bonding between microbial surface amino groups and polydopamine. Under mild alkaline conditions, polydopamine is synthesized by the self-polymerization of dopamine to create a protective coat on a microbial cell surface (Yang et al., 2011). The thickness of polydopamine self-polymerized layers can be well-controlled by repetitive coating to enhance the protection offered to encapsulated microorganisms, but this goes at the expense of metabolite exchange and growth. Alike layer-by-layer self-assembled shells, polydopamine layers can also be employed as a template for further modification and grafting new functionalities to direct environmental interactions (Su et al., 2019).

Recently, adamantane receptors have been grafted on the surface of *Clostridium butyricum* to create ligand-receptor binding of β-cyclodextrin (ligand) modified dextran to create an encapsulating shell (Zheng et al., 2020).

### Inorganic Encapsulating Materials

**Figure 2:**
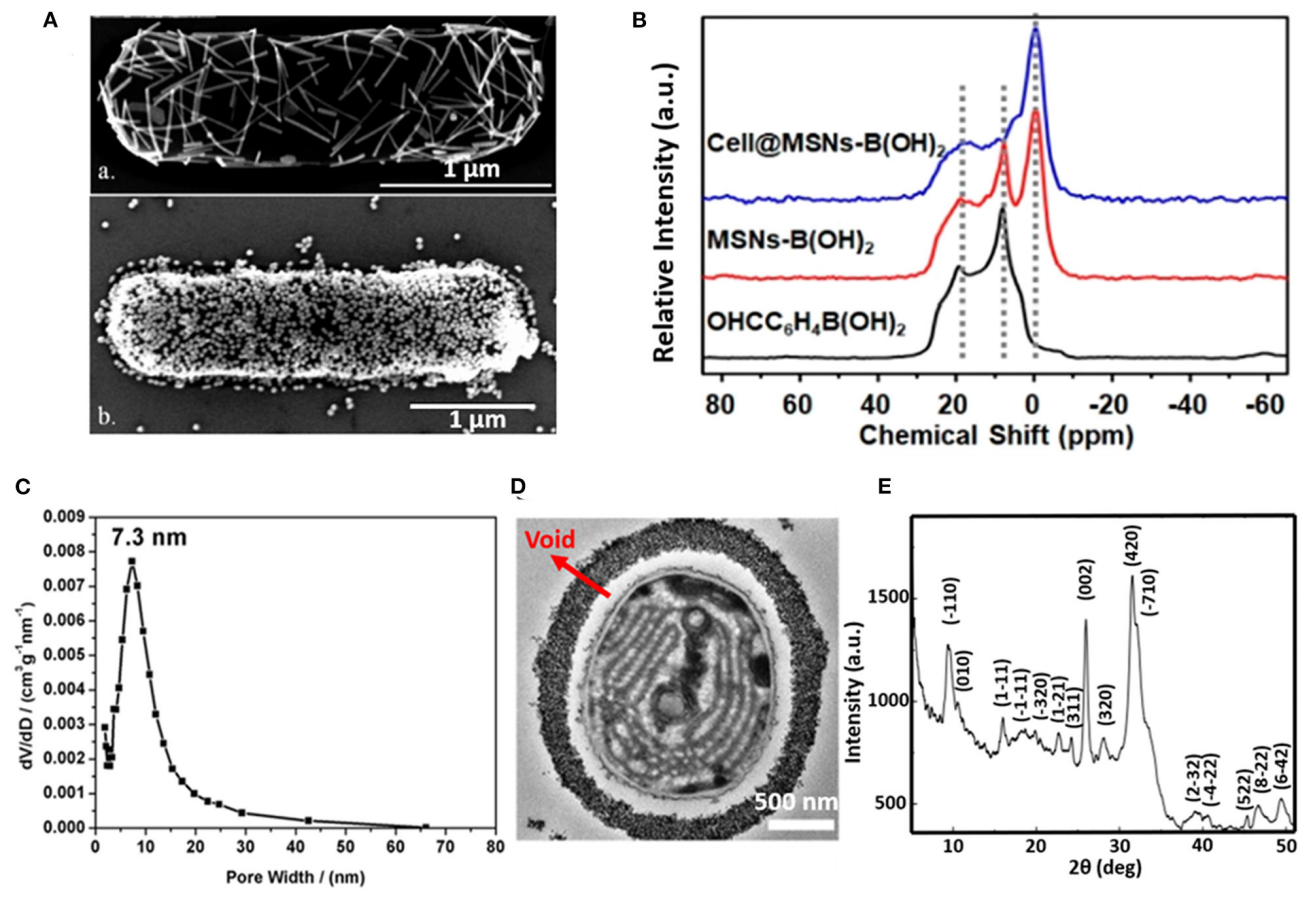
Examples of characteristics of different inorganic, surface-engineered shells. **(A)** SEM micrograph of *B. cereus* encapsulated by directly deposited CTAB terminated Au nanorods (upper image) and Au nanoparticles (bottom image) (Berry et al., 2005, copyright 2005, American Chemical Society). **(B)** ^11^B NMR spectra of 4-formylphenylboronic acid (black line), B(OH)_2_ modified mesoporous SiO_2_ nanoparticles (red line), demonstrating binding of B(OH)_2_ modified SiO_2_ mesoporous nanoparticles to *S. cerevisae* surfaces (blue line) (Geng et al., 2019, copyright 2019, American Chemical Society). **(C)** Pore size distribution of the SiO_2_ yolk-shell encapsulated cyanobacteria *Synechocystis* sp. PCC 7002, determined by analysis of nitrogen adsorption-desorption isotherms (Wang et al., 2020, copyright 2020, Oxford University Press). **(D)** TEM micrograph of a cyanobacterium encapsulated by a SiO_2_ yolk shell, showing a void between the shell and the bacterial cell surface characteristic to a yolk-shell (Wang et al., 2020, copyright 2020, Oxford University Press). **(E)** XRD pattern of a calcium phosphate biomineralized shell encapsulating *S. cerevisiae* after layer-by-layer application of PDADMAC and PAA to initiate precipitation and mineralization (Wang et al., 2008, copyright 2008, Wiley-VCH).

The silica exoskeleton of diatoms and egg shells provide examples of naturally occurring encapsulating shells. This has inspired the use of silica and other materials for microbial encapsulation (see Table 1). Direct deposition of inorganic encapsulating materials is usually achieved through electrostatic double-layer attraction between negatively charged microbial surfaces (Fakhrullin et al., 2012) and inorganic nanoparticles. Often this requires a cationic coating of the inorganic nanoparticle. Positively charged hexadecyltrimethylammonium bromide (CTAB) terminated Au nanorods and nanoparticles have been deposited on *Bacillus cereus* cell surfaces through electrostatic double-layer attraction (Figure 2A) (Berry et al., 2005), while inorganic graphene nanosheets modified with Au-Ca^2+^ nanoparticles have been deposited on the surfaces of *S. cerevisiae* yeasts for application in biosensors (Kempaiah et al., 2011).

Phenolboronic acid based click-reaction chemistry has been applied for the reversible encapsulation of yeasts, that possess a large number of cis-diol containing polysaccharides on their cell surface (Geng et al., 2019). To this end, mesoporous SiO_2_ nanoparticles were modified to expose B(OH)_2_ groups and bind to hydroxyl groups on a yeast surface (Figure 2B). The accumulation of SiO_2_ nanoparticles resulted in a uniform shell with a high porosity (Figure 2C). As a unique feature of this type of binding, encapsulation can be made undone by the addition of glucose.

In yolk-shell encapsulation (Wang et al., 2020) a cell-penetrating peptide is used to create a temporary, cationic coating on a microbial cell surface that can bind negatively charged silica nanoparticles. The cationic coating slowly disappears due to internalization of the peptide into the cell, leaving a void characteristic for yolk-shell encapsulation (Figure 2D). Due to the lack of direct contact between the shell and the cell surface, yolk-shell encapsulation yields long-term viability of encapsulated cells.

Biomineralization involves *in situ* synthesis of an inorganic shell around a microbial cell. However, microbial cell surfaces are generally unsuitable for inducing spontaneous mineralization due to lack of interaction between microbial cell surface components and precursors required to initiate biomineralization. Therefore, in a first step, the microbial cell surface needs to be modified to initiate precipitation and biomineralization. Layer-by-layer treatment of *S. cerevisae* using poly(diallyldimethylammoniumchloride) (PDADMAC) and poly(acrylic sodium) (PAA) effectively bound Ca^2+^ ions from a calcification solution for biomineralization (Figure 2E) (Wang et al., 2008) and initiated biomineralization of silica (Yang et al., 2009). As an alternative for polyelectrolytes, peptide coatings have also been employed to initiate precipitation and mineralization of TiO_2_ (Yang et al., 2012) and SiO_2_-TiO_2_ composite materials (Ko et al., 2013). Biomineralization can also be performed without a polyelectrolyte or peptide template by using biodegradable MnO_2_ nanozymes through Mn-based biomineralization (Li et al., 2017).

### Organic-Inorganic Hybrid Encapsulating Materials

**Figure 3:**
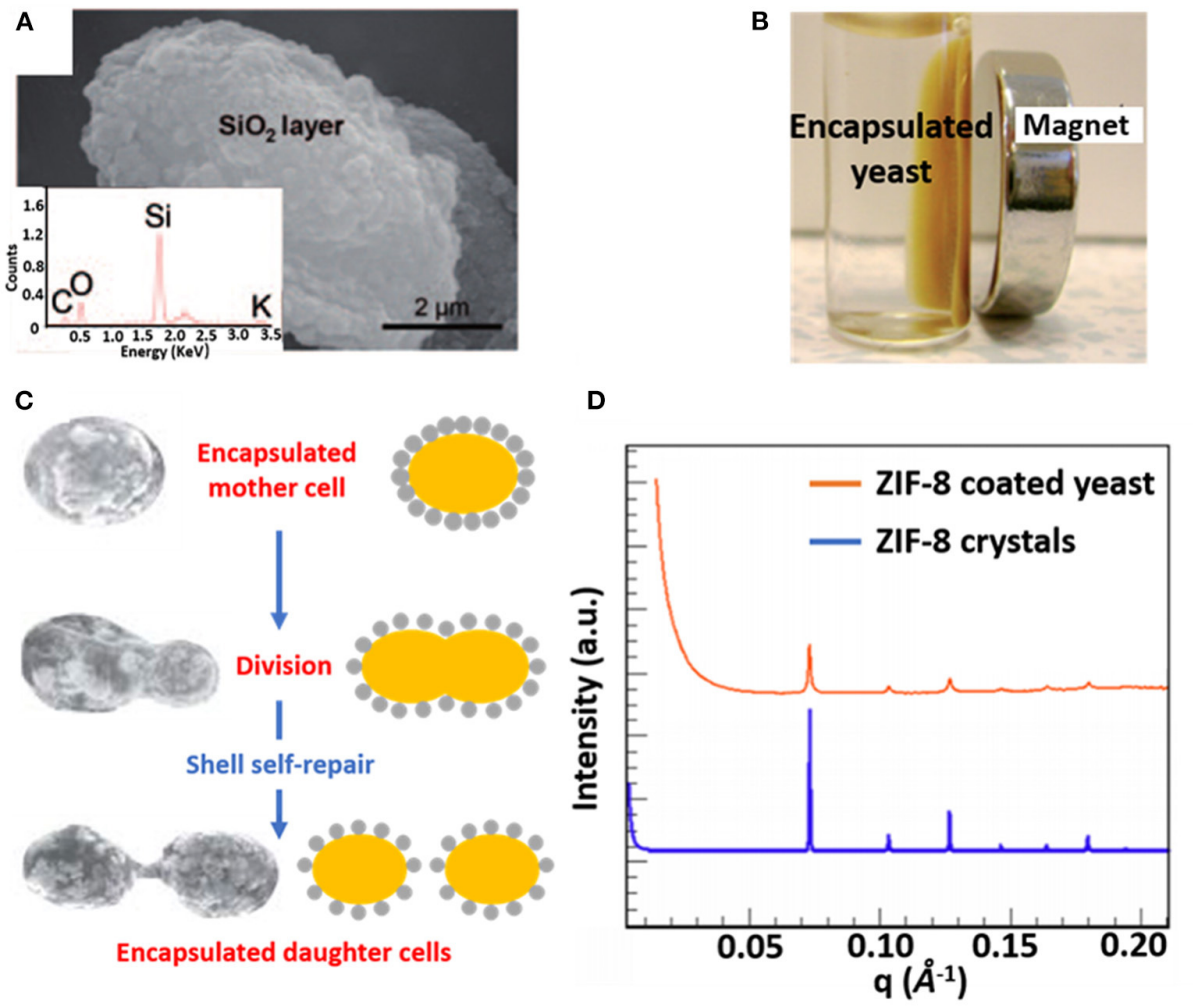
Examples of characteristics of different organic-inorganic, surface-engineered shells. **(A)** SEM micrograph and an Energy-dispersive X-ray line-scan of *S. cerevisiae*, encapsulated with a hybrid shell, composed of PDADMAC-PAA layers and silica nanoparticles (Wang et al., 2010, copyright 2010, Wiley-VCH). **(B)** Magnetic Fe_3_O_4_ nanoparticles allow magnetic separation of encapsulated *S. cerevisiae* cells from suspension (Fakhrullin et al., 2010, copyright 2010, The Royal Society of Chemistry). **(C)** Self-repair of biohybrid nanoshells composed of L-cysteine functionalized Au nanoparticles around *S. cerevisiae* upon division of an encapsulated mother cell (Jiang et al., 2015b, copyright 2015, The Royal Society of Chemistry). **(D)** Small-angle X-ray scattering (SAXS) diffraction pattern of ZIF-8 encapsulated *S. cerevisiae*, demonstrating a crystalline structure similar as observed in ZIF-8 crystals (Liang et al., 2016, copyright 2016, WILEY-VCH).

Combining organic and inorganic materials offers more flexibility in functional design of an encapsulating shell than the use of purely organic or inorganic shells. Hybrid shells composed of a combination of organic and inorganic materials have been described for polyelectrolyte layer-by-layer coatings combined with silica nanoparticles (Figure 3A) (Wang et al., 2010), carbon nanotubes (Zamaleeva et al., 2010) or magnetic Fe_3_O_4_ nanorods (Fakhrullin et al., 2010). Encapsulation with magnetic nanorods yields the added feature of allowing magnetic separation of encapsulated microorganisms (Figure 3B).

Hybrid shells composed of L-lysine modified nanoparticles have been applied to encapsulate *Synechococcus* with self-assembling silica nanoparticles resulting in a mesoporous shell (Jiang et al., 2015a). Similarly, L-lysine modified Au nanoparticles have been used to encapsulate desulfurizing *Gordonia* sp. with a protective shell (Jiang et al., 2014). Self-assembly of the modified nanoparticles on microbial cell surfaces was initiated through hydrogen bonding between amino and carboxyl groups on the nanoparticles and hydroxyl and amino groups on the microbial cell surfaces, respectively. Hybrid shells possessing Au nanoparticles have also been applied to encapsulate *S. cerevisiae*, exhibiting the interesting phenomenon of “self-repair,” implying encapsulation of daughter cells after growth and separation from a mother cell (Figure 3C) that effectively lasted 4–5 generations (Jiang et al., 2015b).

Metal–organic frameworks, including ZIF-8 (Liang et al., 2015) and TA–Fe^III^ (Park et al., 2014) have also emerged as encapsulating materials. When applied to *S. cerevisiae* surfaces ZIF-8 shells demonstrated a highly porous and crystalline structure (Figure 3D) (Liang et al., 2015), while both ZIF-8 (Liang et al., 2015) and TA–Fe^III^ (Park et al., 2014) shells can be degraded on-demand to control the growth of encapsulated cells.

### Surface Characterization of Encapsulated Microorganism

Common methods applied in the characterization of surface-engineered shells encapsulating microorganisms, encompass traditional methods applied in physico-chemistry. Some methods can be applied to encapsulated microorganisms in their natural hydrated state (zeta potentials), others require extensive (freeze-)drying like Scanning Electron Microscopy (SEM), Transmission Electron Microscopy (TEM), X-ray Diffraction (XRD), Fourier Transform Infrared Spectroscopy (FTIR), Nuclear Magnetic Resonance spectroscopy (NMR) or nitrogen adsorption-desorption isotherms. One of the main advantages of zeta potential measurements (particulate microelectrophoresis) is that the data are obtained in a hydrated state, natural to microorganisms in most applications. Moreover, zeta potentials reflect the properties of the outermost surface of the shells that are directly involved in interaction of encapsulated microorganisms with their immediate environment. Most methods applied for the characterization of encapsulated microorganisms, however, relate to the encapsulating shell, including the interior and exterior of the microorganisms. The depth of information of FTIR and Energy Dispersive X-ray Spectroscopy (EDS) for example, amounts several micrometers, which exceeds the thickness of most surface-engineered shells (Binder et al., 2018; Guentsch et al., 2019). Therewith, information about the surface composition of surface-engineered shells has hardly been provided. Yet, XPS is frequently applied to determine the elemental surface composition of different materials and coatings, including microbial cell surfaces. This makes it surprising that XPS is “*forgotten*” as a characterization method of surface-engineered shells.

## XPS for Microorganism Surface Characterization

XPS has been widely applied for the characterization of material surfaces. XPS quantitatively measures the elemental composition of a surface, including the chemical functionalities in which elements are involved. The probing depth of XPS is ~10 nm, which makes it suitable for analysis of the near-surface region of materials. XPS has been quite popular for the characterization of microbial cell surfaces. Microbial cell surface XPS is relatively simple compared with biochemical analyses, requiring only freeze-drying of the microorganisms under study. Moreover, despite the enormous variety in the microbial world, the number of elements detectable in microbial cell surfaces is generally limited enabling the use of simple interpretative models to describe microbial cell surfaces. In order to “*revive*” XPS as a method for the characterization of microbial cell surfaces and advocate its use for the characterization of surface-engineered shells, we now first present a brief description of microbial sample preparation for XPS and outline of generally applicable interpretative models. Selected examples of XPS characterization of unencapsulated microbial cell surfaces will be given.

### XPS for Microbial Cell Surface Characterization: Sample Preparation

**Figure 4:**
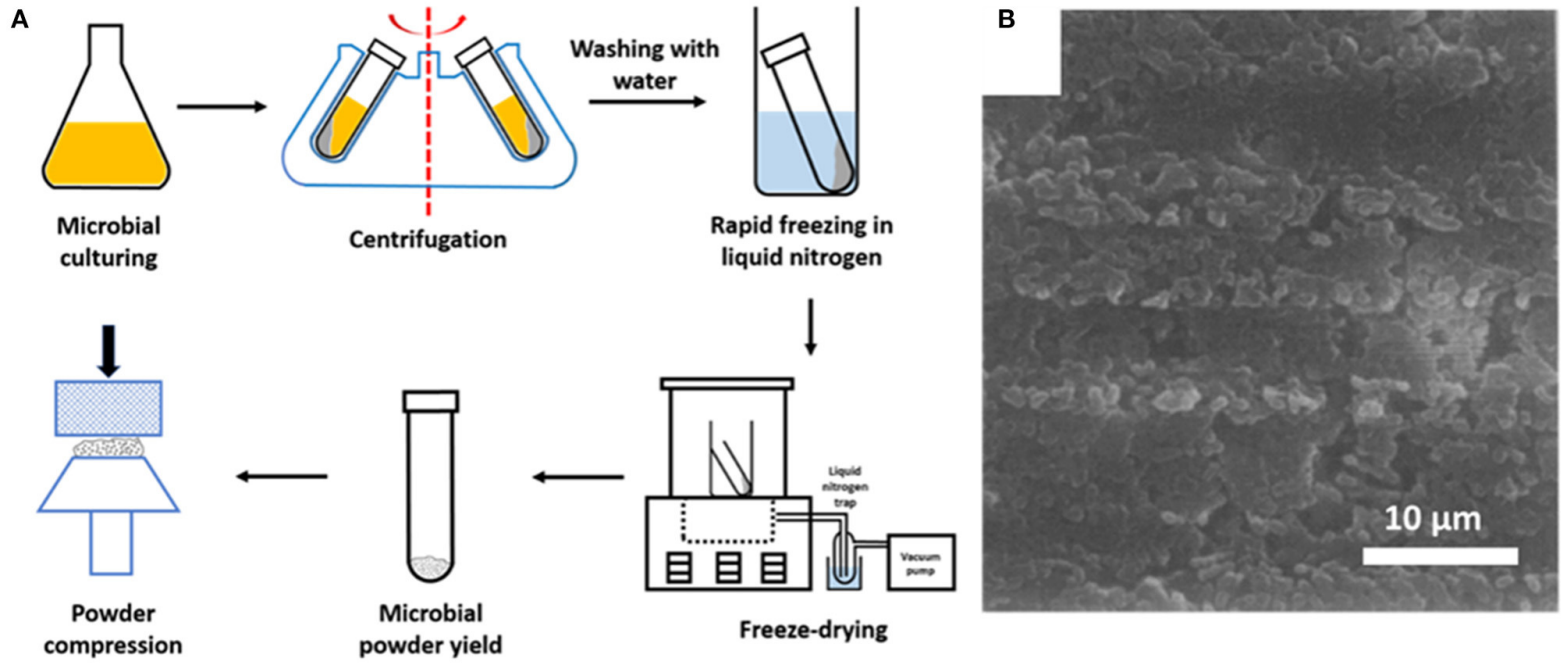
**(A)** Preparation of microbial samples for XPS analyses (adapted from Van der Mei et al., 2000). **(B)** SEM image of the surface of a compressed powder of freeze-dried *Streptococcus salivarius* in a stainless steel cup, suitable for XPS analysis (Van der Mei and Busscher, 1990, copyright 1990, Elsevier Inc).

Microorganisms, cultured in a liquid medium, must first be collected by centrifugation and washed in distilled water to remove medium components from the microbial cell surfaces (Figure 4). Centrifugation and washing are both critical steps. Centrifugation may damage the microbial cell surface (Marshall et al., 1994; Peterson et al., 2012), while washing must be done in water, since washing in more physiological fluids like saline or phosphate buffers yield deposition of Na, Cl, P or O species at the surface that interfere with the determination of the elemental cell surface composition. After washing, microorganisms must be rapidly cooled in liquid nitrogen and subsequently transferred to a freeze-dryer. Freeze-drying bears the danger of carbon contamination, in addition to unavoidable carbon contamination in the XPS, originating from the vacuum pumps employed. Therefore, freeze-drying is recommended to be done in machines equipped with a cold plate or liquid-nitrogen trap to avoid carbon contamination of the surfaces during freeze-drying. For the similar reason of avoiding carbon contamination, samples should be stored *in vacuo* for as short as possible times prior to analyses.

### Chemical Modelling of Microbial Cell Surfaces and Validation

**Figure 5:**
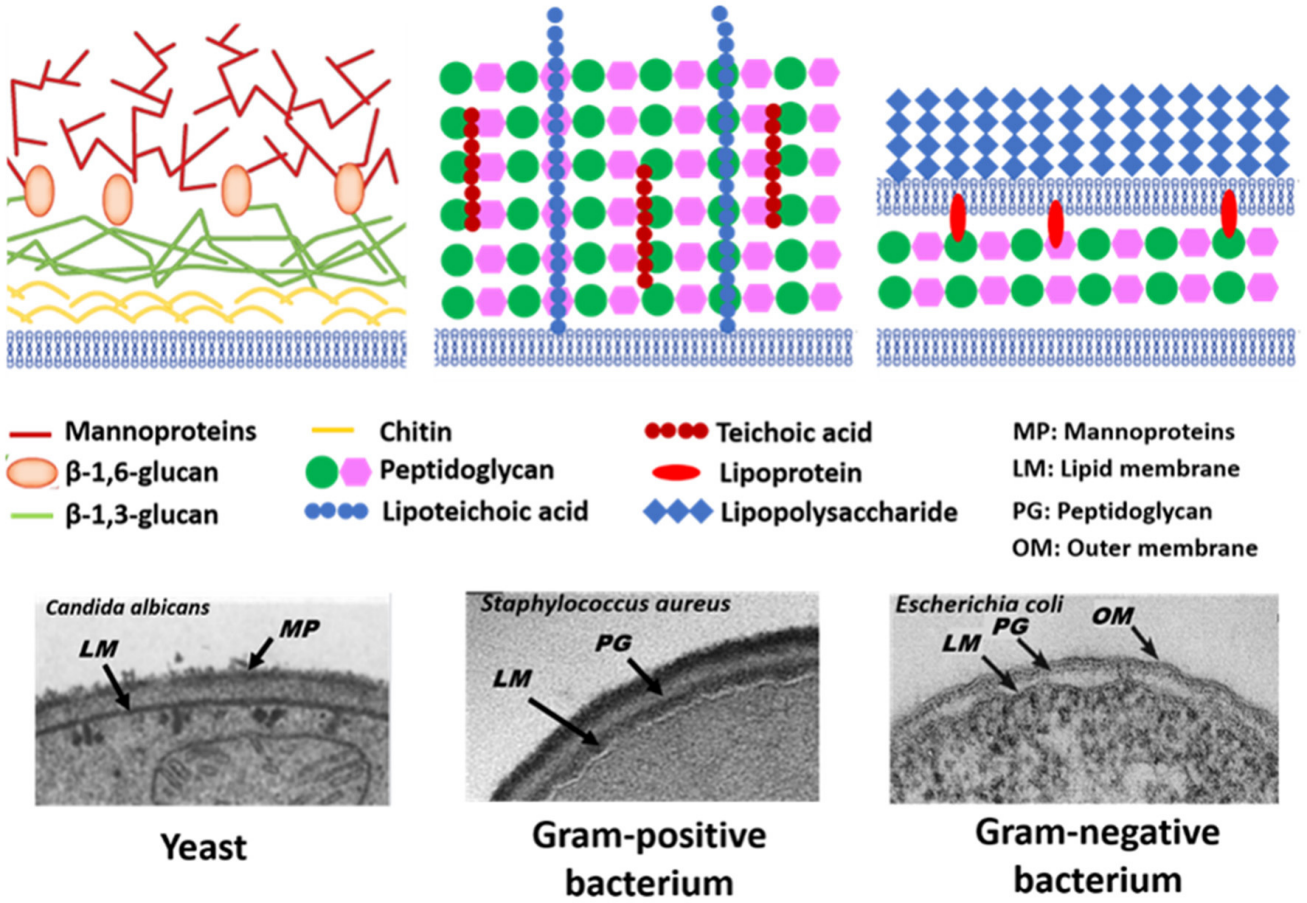
Schematics and TEM micrographs of microbial cell walls, including yeasts, Gram-positive, and Gram-negative bacteria. Panel on *Candida albicans* taken from Hardison and Brown (2012) (copyright 2012, Nature Publishing Group) and Osumi (1998) (copyright 1998, Elsevier Science Ltd.), panel on *Staphylococcus aureus* taken from Boudjemaa et al. (2019) (copyright 2019, Elsevier B.V.) and *E. coli* panel taken from Beveridge (1999) (copyright 1999, American Society for Microbiology).

**Figure 6:**
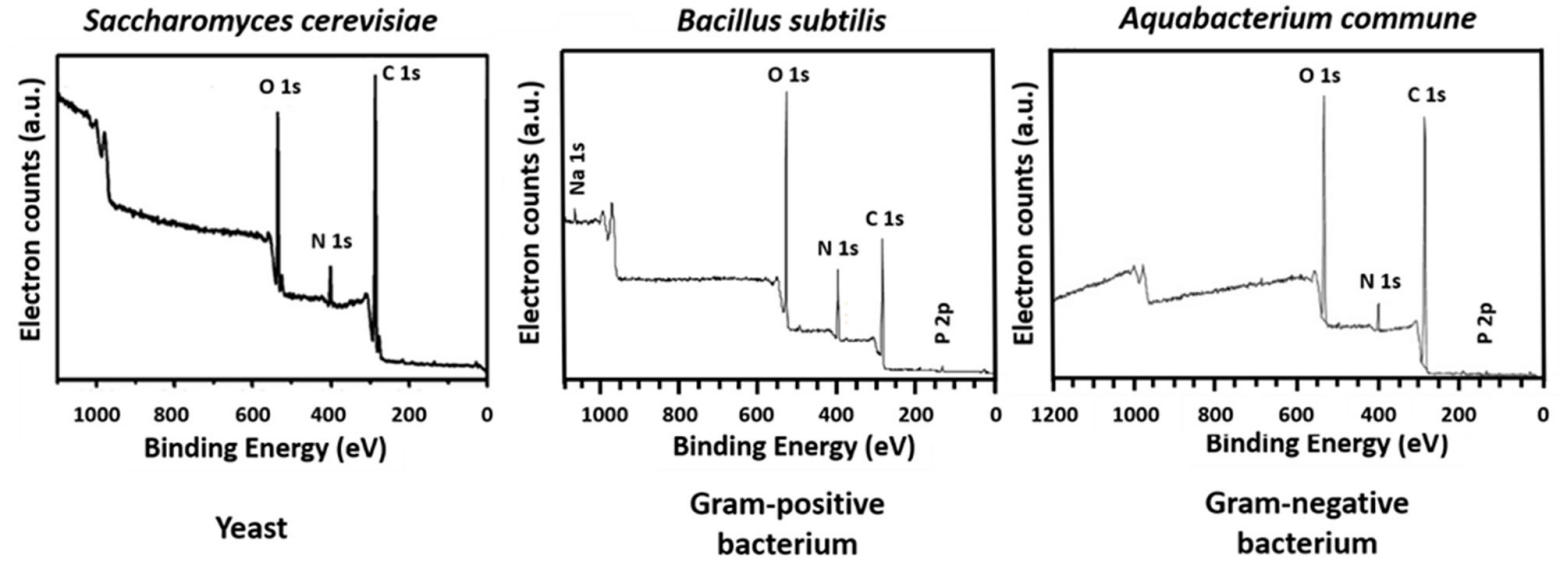
Wide scans of microbial cell surfaces of yeasts, Gram-positive, and Gram-negative bacteria. Panel on *S. cerevisiae* taken from Xia et al. (2015) (copyright 2014, Elsevier B.V.), panel *B. subtilis* taken from Leone et al. (2006) (copyright, 2006, John Wiley and Sons) and panel on *Aquabacterium commune* taken from Ojeda et al. (2008) (copyright 2008, American Chemical Society).

The microbial world consists amongst others of yeasts and bacteria. Yeasts are eukaryotes that distinguish themselves from bacteria by the possession of a nucleus, and growth in warm and moist places possessing the ability to produce alcohol, esters and phenols. The yeast cell wall consists of a cytoplasmic lipid-membrane, a periplasmatic space covered by an outer wall consisting of β-1,3-glucan-chitin complexes and mannoproteins on top of it. The cell wall is mainly composed by β-glucan, β-glucan-chitin and mannoproteins (Figure 5). Bacteria are prokaryotic microorganisms without a nucleus confining their DNA, and can be divided into Gram-positive and Gram-negative strains (Figure 5). The cell wall of Gram-positive bacteria is composed of a thick and rigid peptidoglycan layer, underlying cytoplasmic lipid-membrane. Gram-negative bacteria possess a double membrane, with a thin peptidoglycan layer with a thickness of about 1–2 nm sandwiched between the inner and outer lipid-membrane. Teichoic acids, lipids, proteins and polysaccharides can be attached to the peptidoglycan to form a bacterium's outer cell surface, as arranged in different cell surface structures, also called cell surface appendages. Importantly, whereas these appendages can stick out from a microbial cell surface under physiological conditions, they collapse onto the cell surface after freeze-drying. Wide scan electron binding energy spectra of yeasts and bacteria (see Figure 6), generally show similar elements, although occurring in different ratios and chemical functionalities. The elemental composition and chemical functionalities in which they occur can be applied in interpretative models yielding a description of yeast and bacterial cell surfaces, corresponding with the biochemical components presented in Figure 5.

#### Biochemical Modelling of Yeast Surfaces

Biochemically, the yeast surface can be envisaged (see Figure 5) as being composed of proteins (Pr), glucan (Gl) and chitin (Chi), together with hydrocarbon-like components (Hc). In an interpretative, generalized model of XPS data, it requires four equations to calculate the yeast cell surface composition based on these four components. Four equations can be set up based on the theoretical occurrence of nitrogen and the three functionalities in which carbon can be involved in a yeast surface (see Table 2). Accordingly, these theoretical occurrences can be related with measured XPS data to yield the percentage occurrence of each component according to Gerin et al. (1993).

(1) $$\begin{array}{r} \text{N}/\text{C}=0.270{\text{C}}_{\mathrm{Pr}}\;+\;0.125{\text{C}}_{\text{Chi}} \end{array}$$

(2) $$\begin{array}{r} (\text{C}=\text{O})/\text{C}=0.280{\text{C}}_{\mathrm{Pr}}\;+\;0.250{\text{C}}_{\text{Chi}}\;+\;0.167{\text{C}}_{\text{G}\text{l}} \end{array}$$

(3) $$\begin{array}{r} {}[\text{C}-(\text{O},\text{N})]/\text{C}=0.320{\text{C}}_{\mathrm{Pr}}\;+\;0.625{\text{C}}_{\text{Chi}}\;+\;0.833{\text{C}}_{\text{G}\text{l}} \end{array}$$

(4) $$\begin{array}{r} {}[\text{C}-(\text{C},\text{H})]/\text{C}=0.400{\text{C}}_{\mathrm{Pr}}\;+\;0.125{\text{C}}_{\text{Chi}}\;+\;{\text{C}}_{\text{H}\text{c}} \end{array}$$

in which C_i_ represents the fraction of carbon associated with each component. These fractions can be converted in to weight fractions by using the carbon concentration in each component (see also Table 2). Similar equations can be set up decomposing the O_1s_ electron binding energy peak into different components, but use of the O_1s_ peak was described to yield less consistent results. Since chitin is mostly present underneath protein and glucan layers, it is sometimes assumed to neglect chitin in the model, as it does not occur within the probing depth of XPS. Using this model, dormant spores of *Phanerochaete chrysosporium* were found to be composed for 45 wt% of protein, 20 wt% of glucan, and 35 wt% of hydrocarbon-like compounds, with the amount of protein decreasing and the amount of glucan increasing upon germination of the spores.

**Table 2:** Fraction of different carbon functionalities based on the C_1s_ binding energy peak and N/C elemental surface concentration ratio of different components of yeast cell surfaces, based on their molecular structure (Gerin et al., 1993).

**Cell surface component**	**C-(C, H)/C**	**C-(O, N)/C**	**C=O/C**	**N/C**	**Carbon concentration (mol g^**−1**^)**
Protein	0.400	0.320	0.280	0.270	0.042
Glucan	0	0.833	0.167	0	0.037
Chitin	0.125	0.625	0.250	0.125	0.039
Hydrocarbon-like compounds	1	0	0	0	0.071

#### Biochemical Modelling of Bacterial Cell Surfaces

For bacteria, an interpretative, generalized model has been presented in which the bacterial cell surface is considered to be composed of protein (Pr), peptidoglycan (Pg), teichoic acid (Ta), polysaccharide (Ps), and hydrocarbon-like compounds (Hc) (Rouxhet et al., 1994). Based on the theoretical elemental composition ratios of these components (see Table 3), measured XPS elemental surface concentration ratios with respect to carbon can be expressed in terms of the fractions of Pr, Pg, Ta, Ps, and Hc according to Mozes and Lortal (1995).

(5) $$\begin{array}{r} \text{N}/\text{C}=0.270{\text{C}}_{\mathrm{Pr}}\;+\;0.200{\text{C}}_{\text{P}\text{g}} \end{array}$$

(6) $$\begin{array}{r} \text{O}/\text{C}=0.320{\text{C}}_{\mathrm{Pr}}\;+\;0.500{\text{C}}_{\text{P}\text{g}}\;+\;1.200{\text{C}}_{\text{T}\text{a}}\;+\;0.833{\text{C}}_{\text{P}\text{s}} \end{array}$$

(7) $$\begin{array}{r} \text{P}/\text{C}=0.170{\text{C}}_{\text{T}\text{a}} \end{array}$$

(8) $$\begin{array}{r} 1={\text{C}}_{\mathrm{Pr}}\;+\;{\text{C}}_{\text{P}\text{g}}+{\text{C}}_{\text{T}\text{a}}\;+\;{\text{C}}_{\text{P}\text{s}}\;+\;{\text{C}}_{\text{H}\text{c}} \end{array}$$

in which C_i_ represents the fraction of carbon associated with each component. These fractions can be converted in to weight fractions by using the carbon concentration in each component (see also Table 3). To solve these four equations with five unknowns, the assumption is usually made that the amount of peptidoglycan measured is negligible as it does not occur within the probing depth of XPS after collapse of surface appendages (Dufrêne et al., 1997). The assumption of negligible amounts of peptidoglycan may be avoided by setting up equations in which independent XPS data occur, such as different binding energy components. Application of the model to oral *Streptococcus sanguis* after being bathed in saliva, demonstrated that the wt% of protein on the bacterial cell surfaces increased from 43 to 53 wt%, at the expense of hydrocarbon-like compounds, decreasing from 23 to 16 wt% upon salivary protein adsorption.

**Table 3:** Elemental ratios of bacterial cell surface components, based on their molecular structure (Mozes and Lortal, 1995).

**Cell surface component**	**O/C**	**N/C**	**P/C**	**Carbon concentration (mol g^**−1**^)**
Protein	0.320	0.270	0	0.0424
Peptidoglycan	0.500	0.200	0	0.0410
Teichoic acid	1.00	0.034	0.170	0.0260
Polysaccharide	0.833	0	0	0.0370
Hydrocarbon-like compounds	0	0	0	0.0710

#### Validation of Microbial Cell Surface XPS

**Figure 7:**
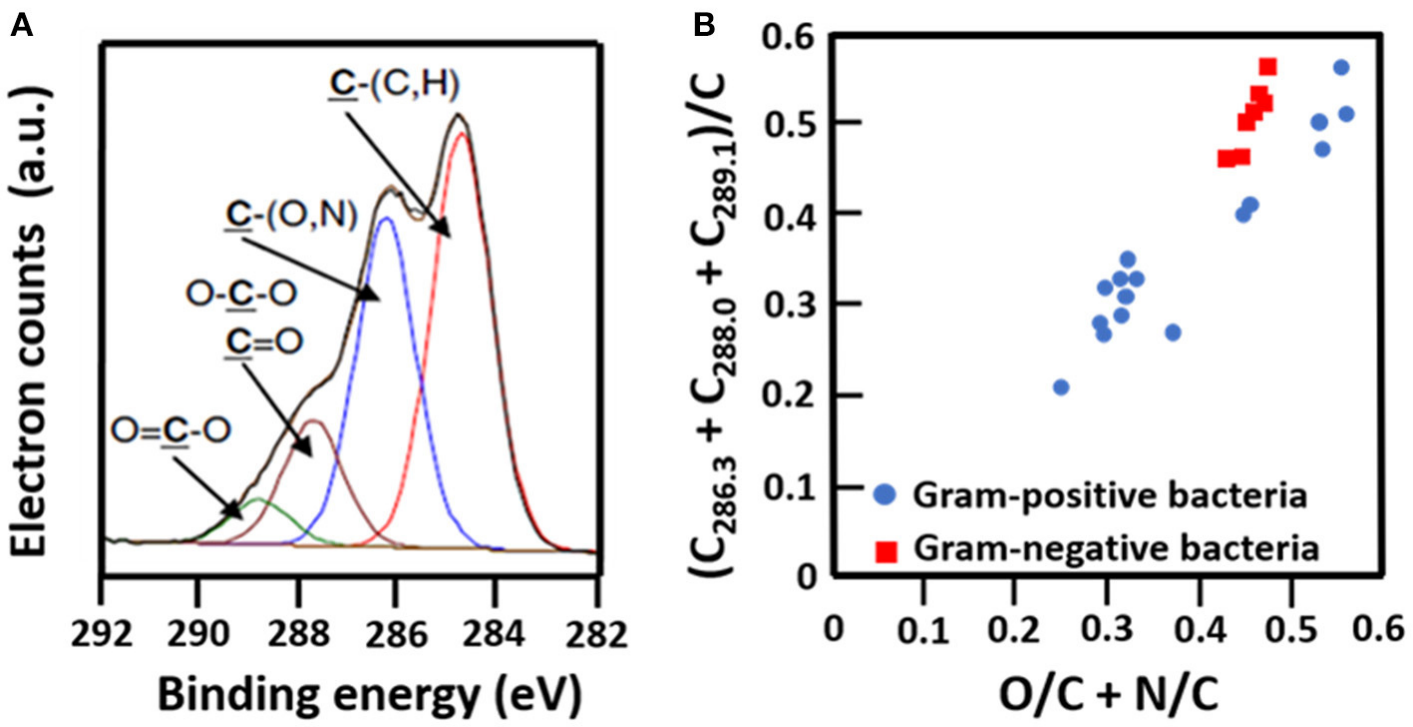
Validation of microbial cell surface XPS, based on a comparison of C_1s_ binding energy components and elemental surface compositions measured. **(A)** Decomposition of the C_1s_ binding energy peak of *B. subtilis* into four components (Ahimou et al., 2007, copyright 2007 Elsevier Inc.). **(B)** The fraction of carbon in bacterial cell surfaces bound to oxygen or nitrogen measured by XPS on a variety of different Gram-positive and Gram-negative strains as a function of the elemental surface concentration of oxygen and nitrogen with respect to carbon (Van der Mei et al., 2000, copyright 2000 Elsevier Science B.V.; Dufrêne et al., 1997, copyright 1997 American Society for Microbiology; Van der Mei et al., 1991, copyright 1991 S. Karger AG. Basel).

**Figure 8:**
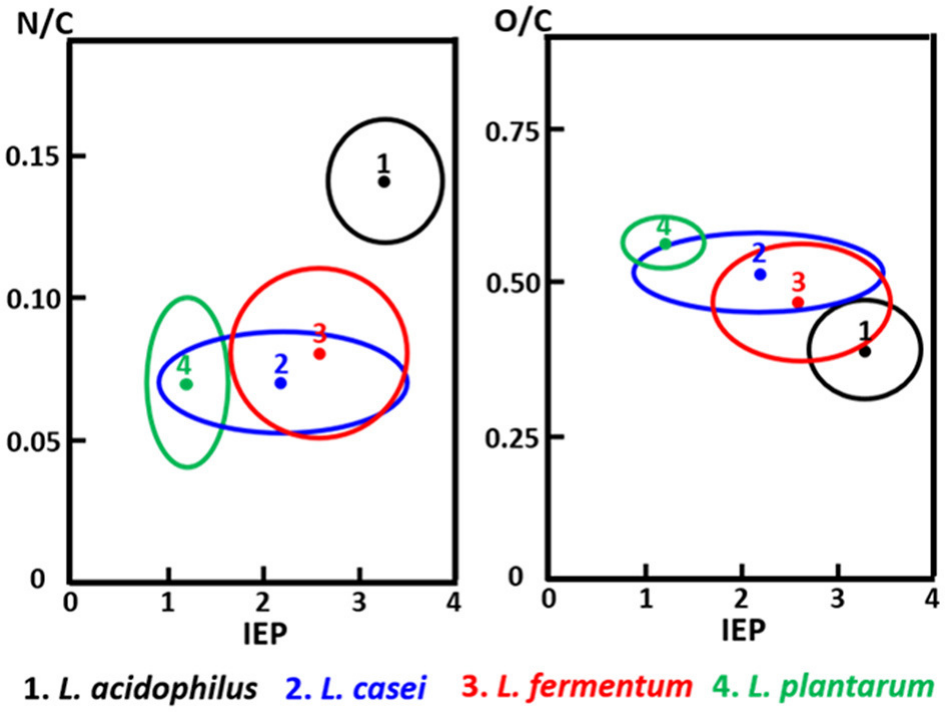
The elemental surface concentration ratios N/C and O/C of Lactobacillus species as a function of their isoelectric points (IEP, pH units). 1, *Lactobacillus acidophilus* (eight strains); 2, *Lactobacillus casei* (eight strains); 3, *Lactobacillus fermentum* (four strains); 4, *Lactobacillus plantarum* (seven strains) (Millsap et al., 1997, copyright 1997 NRC, Canada).

The C_1s_ binding energy peak of bacterial cell surfaces is usually composed of four components due to carbon in C-(C, H) functionalities at 284.8 eV, C-(O, N) functionalities at 286.3 eV, C=O functionalities at 287.9 eV and O=C-OH functionalities at 289.0 eV (see Figure 7A for an example). As an internal validation, independently measured fractions of carbon involved in functionalities comprising oxygen or nitrogen should relate, as has been demonstrated for different collections of bacterial strains and species (see Figure 7B for an example). The internal validation in essence represents an internal consistency check of microbial XPS data, without taking possible artefacts due to freeze-drying and the associated collapse of cell surface structures into account. Particulate microelectrophoresis is extremely suitable for further validation of microbial XPS data because particulate microelectrophoresis measures zeta potentials of microorganisms in a fully hydrated, natural state representing the opposite condition of their freeze-dried state. A summary of available literature data (Van der Mei et al., 1988a,b; Cuperus et al., 1993; Millsap et al., 1997; Van der Mei and Busscher, 1997) demonstrates that bacterial isoelectric points (IEPs) increase with increasing N/C elemental concentration ratios (Figure 8). Nitrogen is a major element constituting the amide functionalities in proteins that become protonated in an acidic environment below their IEP. Similarly, more oxygen in bacterial cell surfaces is accompanied by a decrease in IEP (see also Figure 8), reflecting the low IEP of phosphates and carboxyl functionalities in which oxygen occurs, i.e., predominantly teichoic acids and polysaccharides (Equation 6). Similar considerations have been forwarded for many other strains and species (Van der Mei et al., 1989; Harkes et al., 1992; Van der Mei and Busscher, 1996) and have collectively led to the conclusion that XPS analyses yields meaningful, quantitative data of microbial elemental cell surface compositions.

### Selected Examples of the Use of Microbial XPS

#### Influence of the Surface Composition of Yeasts on Their Flocculation Behavior in Beer Brewing

**Figure 9:**
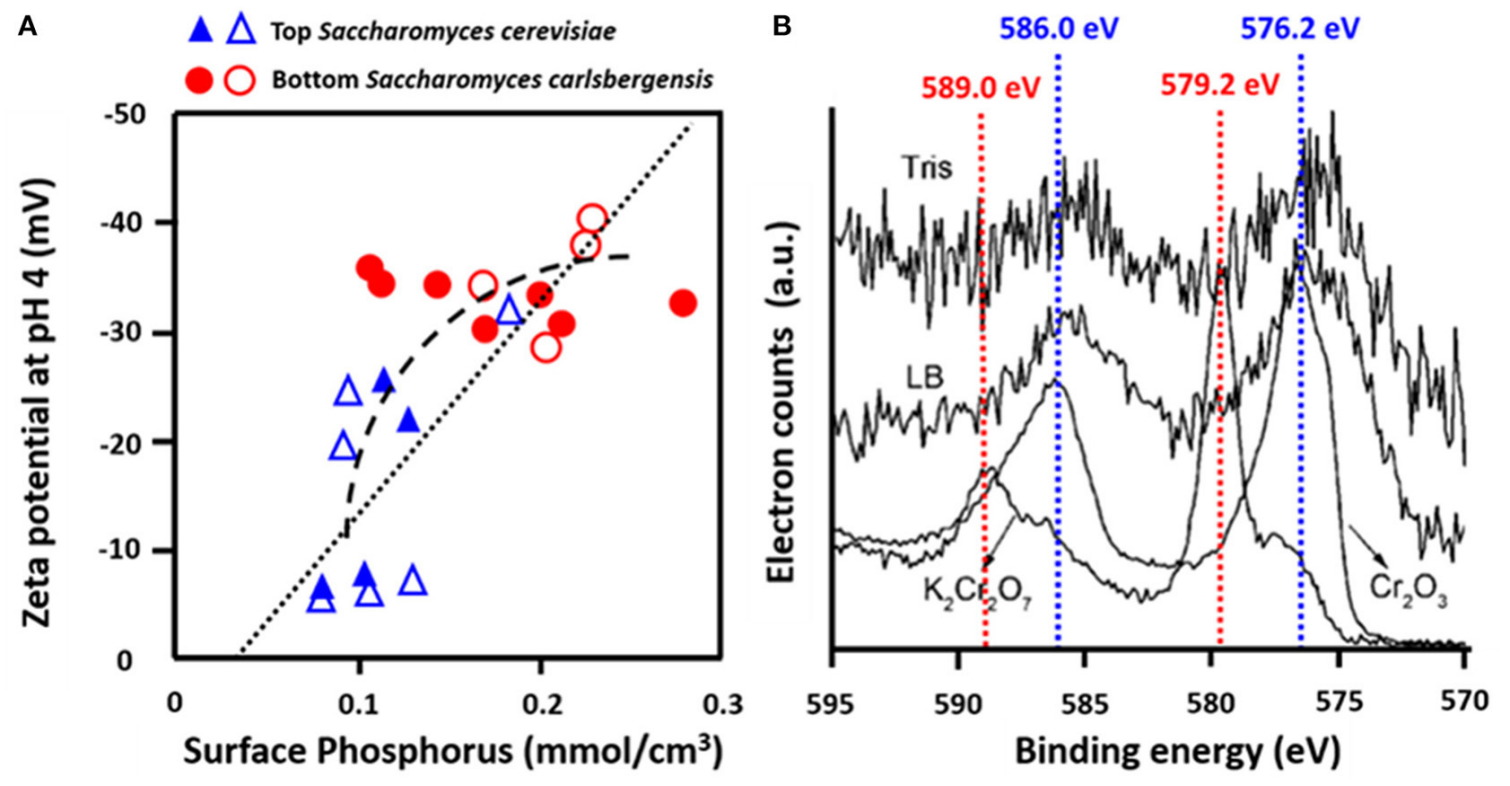
Examples of the use of microbial XPS. **(A)** Zeta potentials of top, *S. cerevisiae* and bottom, *S. carlsbergensis* fermenting yeast strains as a function of the concentration of phosphorus, measured using XPS (Amory and Rouxhet, 1988a, copyright 1988 Elsevier Science Publishers B.V.; Amory and Rouxhet, 1988b, copyright 1999-2021, John Wiley and Sons, Inc.) **(B)** Determination of the valency of Cr adsorbed to *O. anthropic* from Cr_2p_ binding energy peaks in Tris-HCl buffer (Tris) or LB medium (LB) as compared with spectra of Cr (III) in Cr_2_O_3_ and Cr (VI) in K_2_Cr_2_O_7_ (Li et al., 2008, copyright 2008, American Chemical Society).

Yeasts are essential in beer brewing, but many lager and ale beers require removal of flocs of yeasts at the end of the fermentation process. Flocculation of yeasts can occur on the top or bottom of the growth medium (Dengis and Rouxhet, 1997). Top and bottom fermenting yeasts differ in their cell surface composition as measured with XPS. Top fermenting *S. cerevisiae* possessed less phosphorus relative to nitrogen (N/P > 12) than bottom fermenting yeasts (N/P < 10), corresponding with more negative zeta potentials of bottom fermenting *S. carlsbergensis* (Figure 9A) (Amory and Rouxhet, 1988b). Higher N/P ratios reflect a higher amount of mannoproteins with a relatively high IEP and a lower amount of phosphate functionalities with a low IEP, explaining the more positive zeta potentials of top fermenting yeasts.

#### Influence of Microbial Cell Surface Composition in Biosorption

Many bacterial strains have the ability to adsorb heavy metals and volatile organic compounds, providing a low-cost way of bio-purification (Rene et al., 2015). Strain specific differences in the adsorptive capacity have been related to the elemental surface composition of the bacterial cell surfaces as derived from XPS. *Ophiostoma stenoceras* had a lower oxygen surface concentration than *Pseudomonas veronii*, leading to higher adsorption of apolar substances (Cheng et al., 2019). XPS was also applied to demonstrate that Cr more readily adsorbed to the surfaces of *Ochrobactrum anthropi* in its Cr(III) rather than its Cr(VI) state (Figure 9B) (Li et al., 2008). Cr adsorption to *Aeribacillus pallidus* decreased the C_1s_ binding energy components at 286.0 eV (C-O) and 287.8 eV (C=O, O-C=O), indicating involvement of these functionalities in the coordination with Cr (Ma et al., 2019). Similar observations have been made with respect to *Shewanella loihica* PV-4 (Wang et al., 2017), brown seaweed (Park et al., 2008), *Pseudoalteromonas* sp. (Li et al., 2016), and *Leifsonia* sp. (Ding et al., 2018).

## Application of XPS in Characterising Microorganisms Encapsulated by Surface-Engineered Shells

Examples of the scarce use of XPS in characterizing surface-engineered shells around microorganisms are presented below to stimulate more wide-spread use of XPS to this end.

### Encapsulation of Probiotic *Bifidobacterium breve* by Protein-Assisted Nanoparticle Packing

**Figure 10:**
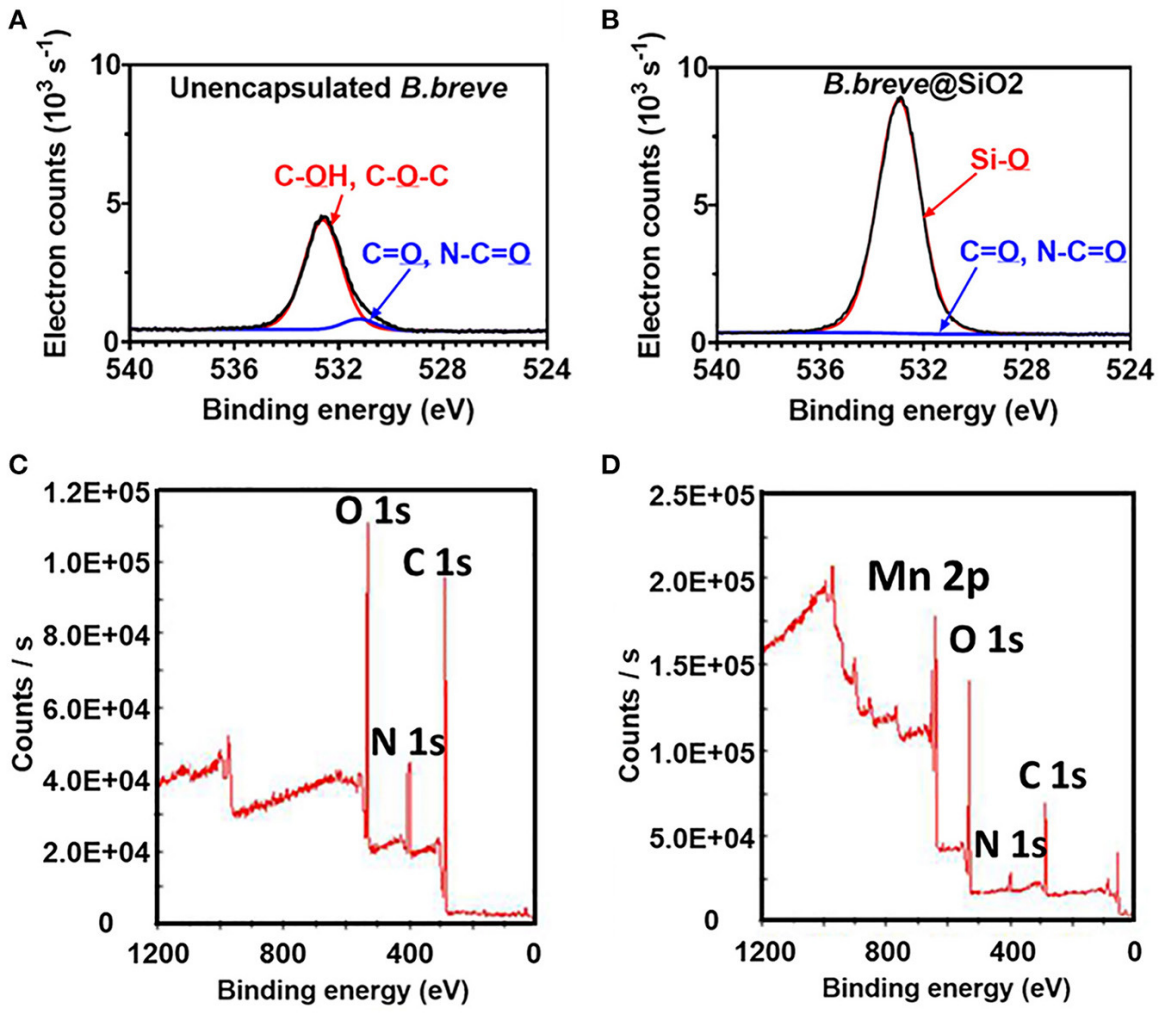
XPS photoelectron binding energy spectra of microorganisms before and after encapsulation. **(A)** Narrow-scan O_1s_ photoelectron binding energy spectra of unencapsulated *B. breve* and **(B)** *B. breve* encapsulated by protein-assisted SiO_2_ nanoparticle packing (Yuan et al., 2021, copyright 2021, American Chemical Society). **(C)** Wide-scan of the photoelectron binding energy spectra of unencapsulated *S. cerevisiae* (Note absence of a Mn_2p_ electron binding energy peak) and **(D)** *S. cerevisiae* encapsulated by a MnO_2_ nanozyme shell (Li et al., 2017, copyright 2017, Wiley–VCH).

Unencapsulated *B. breve* cell surfaces were found rich in carbon (64.4 at%) and oxygen (32.1 at%) using XPS (Yuan et al., 2021), while possessing, 2.1 at% of nitrogen and 0.2 at% of phosphorus. Applying the interpretative model of XPS data for bacteria (Equations 5–8), it can be calculated that the *B. breve* surface is composed for 12.7 wt% out of protein, 63.2 wt% of polysaccharide, 3.1 wt% teichoic acid, and 21.0 wt% of hydrocarbon-like compounds. The presence of a SiO_2_ nanoparticle shell around probiotic *B. breve* could be clearly evidenced using XPS (Yuan et al., 2021) from an increase in the at% O from 32.1 to 59.5%, concurrent with a decrease in at% N from 2.1 to 0.6%. Narrow-scan binding energy spectra of O_1s_ for unencapsulated and encapsulated *B. breve* clearly showed involvement of oxygen in different chemical functionalities present in the cell surface (Figure 10A) and in the SiO_2_ nanoparticle shell (Figure 10B). The decrease in at% N indicates that the nanoparticle shell is relatively thick compared with the depth of probing of XPS, while its SiO_2_ composition is confirmed from a ratio of O:Si (2.06), close to the theoretical ratio of O:Si in SiO_2_.

### Yeasts Encapsulated by Biomimetic Growth of a MnO_2_ Shell

Unencapsulated *S. cerevisiae* cell surfaces (Li et al., 2017) were composed of carbon, oxygen and nitrogen (Figure 10C). The native yeast surface did not contain any Mn and accordingly the presence of a MnO_2_ shell could be easily evidenced by the measurement of Mn in the wide scan electron binding energy spectrum of encapsulated yeast (Figure 10D). Concurrent with the growth of a MnO_2_ shell, the amounts of carbon, oxygen, and nitrogen decreased, similar as observed in nanoparticle packing of *B. breve*.

## Conclusions

XPS is an ideal method for measuring the elemental surface composition of microorganisms and has been applied in widely different fields of application. Preparation of microbial samples suitable for XPS analysis is relatively simple compared with application of XPS to characterize solid materials and coatings, only requiring freeze-drying as an additional step. Comprehensive relationships exist between microbial zeta potentials, measured in a fully hydrated state and elemental microbial cell surface composition, despite being measured in a freeze-dried state of the organisms. Nevertheless, XPS is “*forgotten*” in the emerging field of protective, microbial encapsulation. Two examples of the application of XPS to determine the elemental surface composition of encapsulating shells are presented to stimulate collaboration between XPS and encapsulation experts to advance the important field of microbial encapsulation with surface-engineered shells.

## Author Contributions

All authors have contributed to collection of the literature employed in this review and writing of the text.

## Conflict of Interest

HB is also director of a consulting company, SASA BV (GN Schutterlaan 4, 9797 PC Thesinge, Netherlands). The remaining authors declare that the research was conducted in the absence of any commercial or financial relationships that could be construed as a potential conflict of interest.
